# Association of Daily Exposure to Air Pollutants with the Risk of Tuberculosis in Xuhui District of Shanghai, China

**DOI:** 10.3390/ijerph19106085

**Published:** 2022-05-17

**Authors:** Ying Xiong, Meixia Yang, Zhengzhong Wang, Honglin Jiang, Ning Xu, Yixin Tong, Jiangfan Yin, Yue Chen, Qingwu Jiang, Yibiao Zhou

**Affiliations:** 1 School of Public Health, Fudan University, Building 8, 130 Dong’an Road, Shanghai 200032, China; 20211020140@fudan.edu.cn (Y.X.); 20211020023@fudan.edu.cn (Z.W.); hljiang20@fudan.edu.cn (H.J.); 21111020043@m.fudan.edu.cn (N.X.); 21211020016@m.fudan.edu.cn (Y.T.); 21211020154@m.fudan.edu.cn (J.Y.); jiangqw@fudan.edu.cn (Q.J.); 2 Key Laboratory of Public Health Safety, Fudan University, Ministry of Education, Building 8, 130 Dong’an Road, Shanghai 200032, China; 3 Center for Tropical Disease Research, Fudan University, Building 8, 130 Dong’an Road, Shanghai 200032, China; 4 Xuhui Center for Disease Control and Prevention, Shanghai 200237, China; 5 School of Epidemiology and Public Health, Faculty of Medicine, University of Ottawa, 451 Smyth Road, Ottawa, ON K1H 8M5, Canada; ychen@uottawa.ca

**Keywords:** air pollutants, time-series analysis, tuberculosis

## Abstract

Previous studies have suggested that air pollutant exposure is related to tuberculosis (TB) risk, but results have not been consistent. This study evaluated the relation between daily air pollutant exposure and TB incidence in Shanghai from 2014 to 2019. Overall, there were four pollutants that were positively related to the risk of new TB cases. After a 5 μg/m^3^ increase, the maximum lag-specific and cumulative relative risk (RR) of SO_2_ were 1.081, (95% CI: 1.035–1.129, lag: 3 days) and 1.616 (95% CI: 1.119–2.333, lag: 0–13 days), while for NO_2_, they were 1.061 (95% CI: 1.015–1.11, lag: 4 days) and 1.8 (95% CI: 1.113–2.91, lag: 0–15 days). As for PM_2.5_, with a 50 μg/m^3^ increase, the lag-specific and cumulative RR were 1.064 (95% CI: 1–1.132, lag: 6 days) and 3.101 (95% CI: 1.096–8.777, lag: 0–21 days), while for CO, the lag-specific RR was 1.03 (95% CI: 1.005–1.057, lag: 8 days) and the cumulative RR was 1.436 (95% CI: 1.004–2.053, lag: 0–16 days) with a 100 μg/m^3^ increase. The associations tended to be stronger in male and elderly patients and differed with seasons. Air pollutant exposure may be a risk factor for TB incidence.

## 1. Introduction

Nowadays, tuberculosis (TB) remains one of the major death causes worldwide; over 1.4 million people die each year due to this infectious disease [1]. According to a global survey by World Health Organization (WHO), China is heavily burdened with tuberculosis and accounts for 8.4% of global TB patients [2]. Thus, risk factors such as smoking, alcohol abuse, diabetes, Human Immunodeficiency Virus (HIV) infection, and air pollution should be limited to alleviate the tuberculosis transmission rate [3].

Ambient air pollution is a major global health risk, increasing the risk of morbidity and mortality from cardiovascular and respiratory diseases [4,5]. Exposure to air pollution can reduce individuals’ defense capability against pathogens. Previous studies showed that fuel-induced indoor air pollution had a strong connection with increased risk of tuberculosis infection [6,7,8,9]. Some epidemiological studies have explored the relations between air pollution exposure and TB risk, and the study results are inconsistent. A cohort study in Taiwan found that particulate matter with an aerodynamic diameter less than 2.5 μm (PM_2.5_), carbon monoxide (CO), and nitrogen dioxide (NO_2_) were positively associated with TB risk [10]. A nested case-control study in the United States found that CO and NO_2_ were positively associated with TB risk, but that ozone (O_3_) was inversely associated with TB [11]. However, another cohort study in South Korea found that CO, NO_2_, and O_3_ did not have an association with TB risk, but that sulfur dioxide (SO_2_) did [12]. A similar situation also occurs in short-term time-series studies. Zhu et al. and Huang et al. found that TB risk was positively associated with SO_2_ and O_3_, respectively [13,14]. Nevertheless, some studies have found that SO_2_ and O_3_ are associated with reduced risk [15,16]. Under these circumstances, more research is required to confirm the relationship between air pollutant exposure and TB risk. Published studies have usually examined a 10 μg/m^3^ increase in air pollutants without considering the different ranges of their concentrations. Furthermore, few short-term time-series studies have analyzed the effect of CO. Therefore, our study characterizes the tuberculosis trends according to air pollution variation in the Xuhui district, Shanghai over the period of 2014 to 2019, and assess the association of TB risk and daily exposure to SO_2_, NO_2_, PM_2.5_, CO, and O_3_ by different increments. The modifying effects of gender, age, and season are also evaluated. This study may contribute to the setting of air quality standards and the detection and protection of the potential susceptible population.

## 2. Materials and Methods

### 2.1. Study Area

Shanghai is an eastern city of China, at the mouth of the Yangtze River. Xuhui District is the representative area of the central city of Shanghai, covering an area of 54.93 km^2^. Xuhui District had a population of 1.09 million and a regional GDP of 166.739 billion yuan in 2019. The region has a mild and humid subtropical monsoon climate, short spring and autumn, long winter and summer, abundant sunshine, and abundant rainfall.

### 2.2. TB Incidence

The count of daily new TB cases for the Xuhui District was obtained from the National TB Surveillance System between 1 July 2014, and 31 December 2019. TB cases found in any health institution were reported to this system within 24 h. Pulmonary TB was diagnosed mainly based on the results of pathogen (including bacteriology and molecular biology) examination, as well as a comprehensive analysis of epidemiological history, clinical manifestations, chest imaging, and related auxiliary examinations. The TB cases in this study were newly diagnosed patients including primary and recurrent TB patients. The time of clinical visit was the time of the first visit after the onset of symptoms. The data did not contain any identifiable information. Age, gender, and the date of the clinical visit were available for analysis.

### 2.3. Air Pollutants and Meteorological Factors

The data on air pollutants were collected from the real-time release platform of the air quality of the China Environmental Monitoring Station at Xuhui station, including SO_2_ (μg/m^3^), NO_2_ (μg/m^3^), PM_2.5_ (μg/m^3^), and CO (μg/m^3^) levels for every 24 h and O_3_ (μg/m^3^) levels for every 8 h. Missing values were less than 1%.

The annual average temperature in Xuhui District is 15.5 °C. The winter is cold and dry, often affected by the northwest monsoon from the Mongolian Plateau with a high wind speed. In the summer, the southeast wind often comes from the Pacific Ocean. The average annual precipitation is 1143.1 mm. In the current study, meteorological factor data were provided by surface weather stations through the International Free Exchange of Meteorological Data System, including temperature (°C), wind velocity (m/s), air pressure (mmHg), and relative humidity (%) levels for every 24 h. There was no missing value in meteorological data for the study period of 2014–2019.

### 2.4. Statistical Analyze

Correlations between daily concentrations of air pollutants and meteorological factors were analyzed by the Spearman rank correlation method to explore their relationship. Then, the distributed lag non-linear model (DLNM) was used to assess the association between the daily average concentration of air pollutants and the daily number of new TB cases for a short-term lag time. Poisson regression models were constructed using the Log connection of the Poisson distribution. In addition to air pollutants, the models also contained daily average temperature, wind velocity, and relative humidity. To avoid the effect of collinearity, factors with a correlation coefficient ∣r∣ ≥ 0.7 were excluded from the models [17]. The natural cubic spline function was used to smooth these covariates with a degree of freedom of 3 and adjust the long-term trend with freedom of 6 [13,18]. Other factors, including calendar year, season, and holiday, were set as dummy variables. The natural cubic spline function’s nodes for the exposure-response dimension fitting were set in quintiles. The same function was also used for the exposure-lag dimension fitting, and the lag period was chosen as 21 days [13]. The logarithmic equidistant method provided by the R program package [19] was used for the knots position setting, and a cross-basis function was established for fitting exposure-lag-response:

(1) $$\begin{array}{c} Y_{t}\;\sim \;quasiPoisson\left(\mu_{t}\right)\\ \log\left(\mu_{t}\right)=\alpha +W_{X}^{T}\eta +ns\left(temperature,3\right)+ns(relative\;humidity,3+ns\left(wind\;velocity,3\right)+ns\left(time,6\right)\\ +\lambda year+\delta season+\gamma DOW \end{array}$$

where $Y_{t}$ denotes the actual count of the daily new TB cases and $\mu_{t}$ denotes the expected count; α is the intercept; $W_{X}^{T}\eta$ denotes the cross-basis matrix by using a linear model for each air pollutant; ns denotes the natural cubic splines to control for meteorological factors and time; λ, δ, and γ denote coefficients of the dummy variables for year, season, and day of week (DOW).

The risk of TB incidence was represented by the lag-specific and cumulative relative risks (RRs) estimates and 95% confidence intervals (CIs) per specific increase of air pollutant concentration (per 5 μg/m^3^ increase in SO_2_, NO_2_, and O_3_; per 50 μg/m^3^ increase in PM_2.5_; per 100 μg/m^3^ increase in CO). The reference was set by the class II level of the National Ambient Air Quality Standard (GB3095-2012) to assess the effect of air pollutants. The reference values were 35 μg/m³ for PM_2.5_, 60 μg/m³ for SO_2_, 40 μg/m³ for NO_2_, 0.5 mg/m³ for CO, and 160 μg/m³ for O_3_ (http://www.mee.gov.cn/ywgz/fgbz/bz/bzwb/dqhjbh/dqhjzlbz/201203/t20120302_224165.shtml, accessed on 14 January 2022). The associations were examined overall as well as for different gender (male, female), age (older people: ≥65 years, younger people: <65 years old), and season (warm season: May–October; cold season: November–April) groups. The potential modification effect in subgroups was analyzed by the formula:

(2) $$\left(\hat{Q_{1}}-\hat{Q_{2}}\right)\pm 1.96\sqrt{{\left(\hat{SE_{1}}\right)}^{2}+{\left(\hat{SE_{2}}\right)}^{2}}$$

where $\hat{Q_{1}}$, $\hat{Q_{2}}$ are the point estimates of the RRs for the categories and $\hat{SE_{1}}$, $\hat{SE_{2}}$ represent the corresponding standard errors [20].

The robustness of the model was assessed by sensitivity analyses by (1) building multipollutant models for every single pollutant, (2) modifying the maximum lag time to 19 and 23 days, and (3) changing the degree of freedom from 1 to 5 in meteorological factors.

The analyses were performed by R software version 4.0.3 (https://www.r-project.org/, accessed on 14 January 2022) with the “dlnm” and “splines” packages. The standard of significant results was that the *p*-value was less than 0.05.

## 3. Results

### 3.1. Basic Distributions

There were a total of 1746 new TB cases, with 0.87 cases per day. Among them, 1076 (61.63%) were males and 1316 (75.37%) were <65 years of age. Table 1 presents the averages and quartile distribution of daily air pollutants, as well as meteorological data.

**Figure 1:**
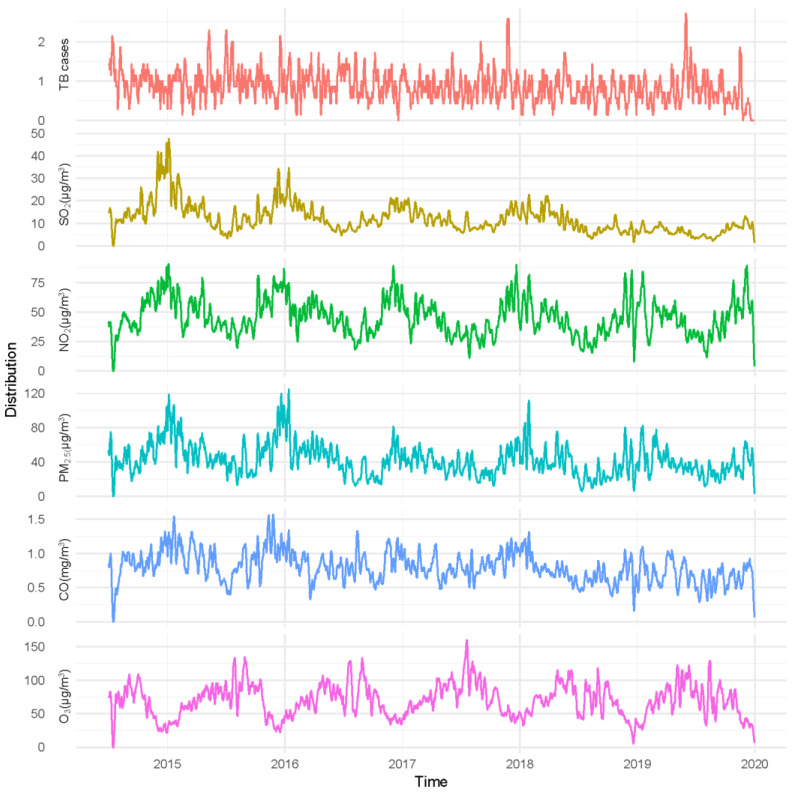
Time series of 7-day moving average number of TB cases and concentrations of air pollutants in Shanghai, China, from 2014 to 2019.

Figure 1 shows the temporal variations of overall daily new TB cases and air pollutants over the study period. The 7-day moving average was used to make the trend more visible. Table 2 showed the correlation coefficients between air pollutants and meteorologic factors. Among meteorological factors, only air pressure and temperature were strongly correlated (r = −0.8861, *p* < 0.05). Other factors were all weakly correlated.

### 3.2. The Relation between Air Pollutant Exposure and TB Incidence

#### 3.2.1. SO_2_

**Figure 2:**
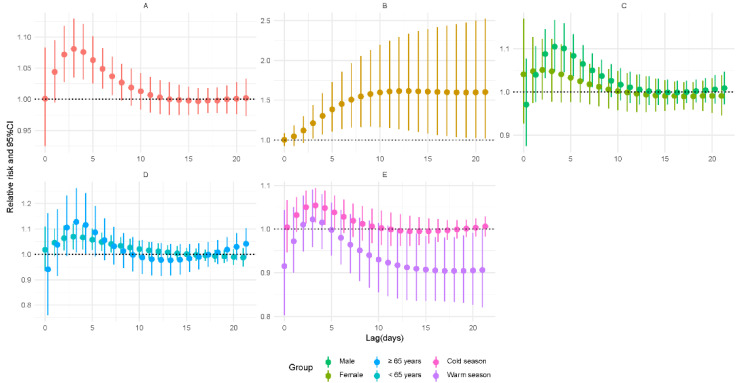
Relative risks for tuberculosis incidence in relation to a 5 μg/m^3^ increase of daily average concentrations of sulfur dioxide (SO_2_). (**A**) Lag-specific RRs; (**B**) cumulative RRs; (**C**) stratified analysis by gender; (**D**) stratified analysis by age; (**E**) stratified analysis by season.

As demonstrated in Figure 2, increased lag-specific risks were found between SO_2_ exposure and TB incidence during 2–7 days of lag. For a lag of 3 days, the RR for a 5 μg/m^3^ expansion was 1.081 (95% CI: 1.035–1.129) overall. The cumulative risk showed an upward trend and reached a maximum on the 13th day of lag (RR = 1.616, 95% CI: 1.119–2.333). In subgroup analyses, the associations were still significant in the male group (RR = 1.105, 95% CI: 1.047–1.167), in both younger (RR = 1.07, 95% CI: 1.02–1.122) and older age groups (RR = 1.128, 95% CI: 1.008–1.262), and in the cold season group (RR = 1.054, 95% CI: 1.015–1.094) on 3-day lag. However, the results of the modification analysis were not significant on 3-day lag (*p* > 0.05) (Table S1).

#### 3.2.2. NO_2_

**Figure 3:**
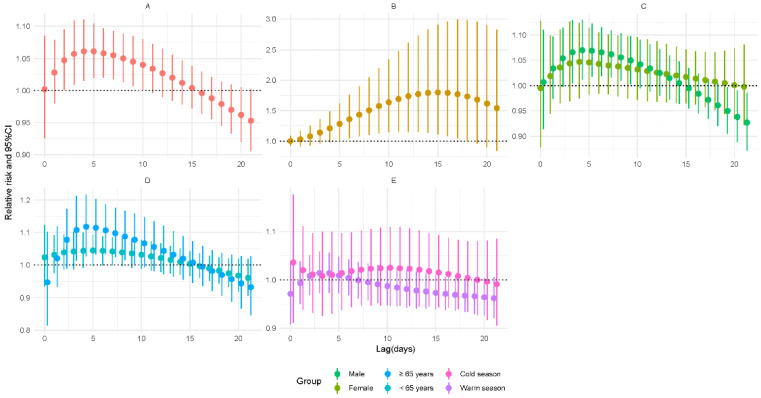
Relative risks for tuberculosis incidence in relation to a 5 μg/m^3^ increase of daily average concentrations of nitrogen dioxide (NO_2_). (**A**) Lag-specific RRs; (**B**) cumulative RRs; (**C**) stratified analysis by gender; (**D**) stratified analysis by age; (**E**) stratified analysis by season.

Figure 3 shows that the risk for TB incidence was positively related to a 5 μg/m^3^ increase in NO_2_ concentration during 2–10 days of lag. On the fourth day of lag, the lag-specific RR was 1.061 (95% CI: 1.015–1.11). The cumulative risk increased during 5–18 days of lag and reached a maximum on the 15th day of lag (RR = 1.8, 95% CI: 1.113–2.91). After stratifying by gender, exposure to NO_2_ raised the risk of male patients (RR = 1.07, 95% CI: 1.012–1.13). As for age, the risk remained significant in older people (RR = 1.118, 95% CI: 1.026–1.218) on a 4-day lag. There was no significant result in season groups. However, the results of the modification analysis were not significant (*p* > 0.05) (Table S2).

#### 3.2.3. PM_2.5_

**Figure 4:**
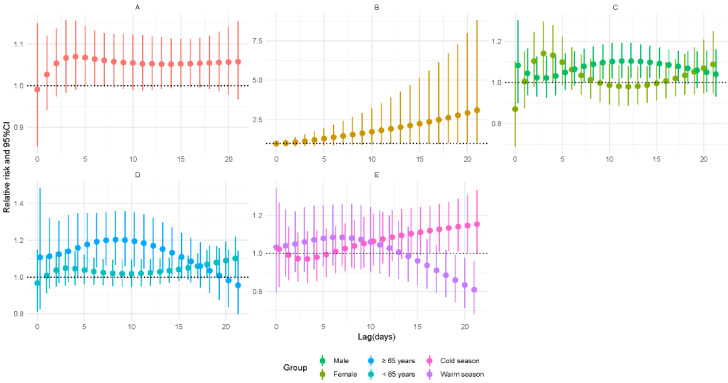
Relative risks for tuberculosis incidence in relation to a 50 μg/m^3^ increase of daily average concentrations of particulate matter with an aerodynamic diameter less than 2.5 μm (PM_2.5_). (**A**) Lag-specific RRs; (**B**) cumulative RRs; (**C**) stratified analysis by gender; (**D**) stratified analysis by age; (**E**) stratified analysis by season.

As demonstrated in Figure 4, for a 50 μg/m^3^ increase, the lag-specific RR of PM_2.5_ was 1.064 (95% CI: 1–1.132) on the sixth day of lag. The cumulative risk increased over time (RR = 3.101, 95% CI: 1.096–8.777). After stratification, the effect remained significant in the male subgroup (RR = 1.104, 95% CI: 1.023–1.191, lag: 12 days). As for age, the older age subgroup showed increased risk after exposure to higher concentrations (RR = 1.203, 95% CI: 1.066–1.358, lag: 8 days). For the season, the cold season group also showed an increased risk (RR = 1.146, 95% CI: 1.006–1.306, lag: 20 days). Only the result of the modification analysis of season was significant (*p* < 0.05) (Table S3).

#### 3.2.4. CO

**Figure 5:**
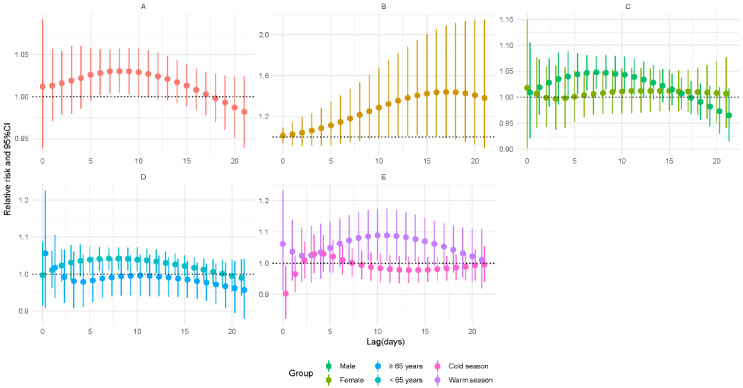
Relative risks for tuberculosis incidence in relation to a 100 μg/m^3^ increase of daily average concentrations of carbon monoxide (CO). (**A**) Lag-specific RRs; (**B**) cumulative RRs; (**C**) stratified analysis by gender; (**D**) stratified analysis by age; (**E**) stratified analysis by season.

Figure 5 shows that the lag-specific RR of 100 μg/m^3^ increase in CO for TB incidence was 1.03 (95% CI: 1.005–1.057) with a lag of 8 days. The cumulative risk increased and peaked on the 16th day of lag (RR = 1.436, 95% CI: 1.004–2.053). Subgroup analyses showed that results were still significant in the male group (RR = 1.048, 95% CI: 1.016–1.08, lag: 7 days), younger age subgroups (RR = 1.042, 95% CI: 1.012–1.072, lag: 8 days), and the warm season (RR = 1.089, 95% CI: 1.009–1.174, lag: 10 days). Only the result of modification analysis of season was significant (*p* < 0.05) (Table S4).

#### 3.2.5. O_3_

**Figure 6:**
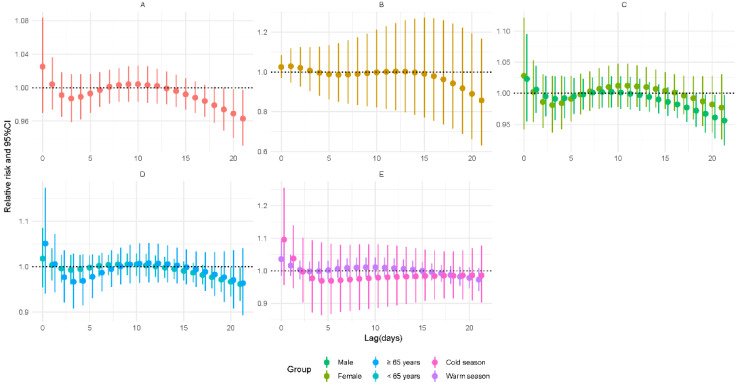
Relative risks for tuberculosis incidence in relation to a 5 μg/m^3^ increase of daily average concentrations of ozone (O_3_). (**A**) Lag-specific RRs; (**B**) cumulative RRs; (**C**) stratified analysis by gender; (**D**) stratified analysis by age; (**E**) stratified analysis by season.

Figure 6 shows that a 5 μg/m^3^ rise of O_3_ was related to a decreased lag-specific risk of TB during the later period (RR = 0.963, 95% CI: 0.931–0.997, lag: 21 days). Subgroup analyses showed that the results remained significant in the male (RR = 0.956, 95% CI: 0.916–0.997) and younger people subgroups (RR = 0.962, 95% CI: 0.925–0.999) on the 21th day of lag. There was no significant result in season groups. However, the modification analysis was not significant (*p* > 0.05) (Table S5).

#### 3.2.6. Sensitivity Analysis

Sensitivity analysis was conducted to assess the stability of the model. First, multipollutant models were built for each single pollutant model. When compared with single pollutant models, there were no obvious changes in multipollutant models (Tables S6–S10). Then, the maximum lag time was modified to 14 and 28 days (Tables S11–S15). Furthermore, the degree of freedom was changed in the meteorological factors (1–5 dfs) (Tables S16–S20). All these results showed that the models were well-fitted and the results were robust.

## 4. Discussion

By employing the DLNM model in time-series analysis, our study found that short-term low levels of SO_2_ and NO_2_ exposure increased TB risk. In contrast, only higher concentrations of CO and PM_2.5_ exposure were positively related to the TB incidence. Short-term O_3_ exposure was not positively related to TB incidence. The stratified analysis showed that SO_2_, NO_2_, CO, and PM_2.5_ exposure was positively related to TB incidence in the male group, NO_2_ and PM_2.5_ exposure was positively related to TB incidence in the elderly group, SO_2_ and PM_2.5_ exposure was positively related to TB incidence in the cold season group, and CO exposure was positively related to TB incidence in the warm season group. Sensitivity analysis demonstrated the robustness of the model.

Our study found that SO_2_ and NO_2_ were positively related to the number of new TB cases within 3–4 days after the exposures. A time-series study showed similar results that NO_2_ and SO_2_ were positively related to the active TB incidence in the first 2 days of the lag in China [13]. Cohort studies, case-control studies, and some long-term time-series studies have also shown that SO_2_ and NO_2_ have a relation to increased risk of TB infection [11,12,21,22]. Other short-term exposure studies have shown that NO_2_ is still associated with an increased risk of TB [15,17]. However, few short-term time-series studies have shown that SO_2_ has a relation to reduced risk of TB [15,16]. The inconsistent results may be caused by the difference in SO_2_ range, and the exposure of cases may not be well presented by a few fixed monitoring stations. The following are the possible mechanisms of the effect of SO_2_ and NO_2_. Short-term exposure to SO_2_ is related to increased pro-inflammatory response. The association between interleukin-6 (IL-6) and SO_2_ showed a short-term lag effect, which was the greatest in the lag of 3 to 5 days [23]. SO_2_ exposure could cause damage to alveolar macrophages, reducing the production and deliverance of tumor necrosis factor-α (TNF-α) [24]. TNF-α and IL-6 have a great influence on the host’s defense against mycobacteria [25]. An experimental study on mice found that SO_2_ inhalation damages the antioxidant defense system in the lungs by reducing the activities and contents of metabolic enzymes in the lung [26]. NO_2_ is a regular air pollutant generally produced by combustion [27]. NO_2_ exposure was associated with inhibition of neutrophil pulmonary infiltration by reducing pulmonary expression of CD62 [28]. NO_2_ exposure enhanced epithelial damage, airway inflammation, hyper-responsiveness, and functional changes in the lung of rodents [27,29,30,31,32]. However, the biological mechanisms for SO_2_ and NO_2_ in relation to tuberculosis risk are still unclear and need further study.

An increased TB risk was observed with a large amount of PM_2.5_ exposure. Some previous studies have indicated that long-term exposure to particulate matter is significantly related to TB risk in China [21,33,34]. Most short-term time-series studies have similar results: that PM_2.5_ has a positive correlation with TB risk [14,15,17]. Several potential mechanisms for particulate matter affecting tuberculosis infection have been discussed. PM exposure could induce cellular senescence and the deregulate expression of antimicrobial peptides of the respiratory alveolar type II epithelial cells [35]. Hence, the intracellular proliferation of Mycobacterium tuberculosis could be increased [36]. Inhalation of PM could also damage human host immune cells (human bronchoalveolar lavage cells, alveolar macrophages, and human peripheral blood monocytes) and inhibit the production of induced interferon γ (IFN-γ), TNF-α, and IL-6, thus weakening the protective antimycobacterial host immunity [25,37,38,39]. Besides, chronic exposure to PM could impair lung redox metabolism and immunity [40].

CO exposure seemed to raise the risk of TB. A case-control study in the United States and a long-term time-series study in China also revealed similar results [11,21]. One short-term time-series study found no significant association [14]. Compared with our study, the increment (10 μg/ m^3^) that this study examined was small, and this may be the reason for the inconsistent results Heme oxygenase produces CO, which was found to have a relation to the inflammatory response [41].

Our study found that ozone was negatively related to the lag-specific risk of TB in our study on the 21st day of lag, but the cumulative risk was not significantly associated. The effect of O_3_ may appear after a lag longer than 21 days, which was in accordance with some previous studies on long-term effects [10,11,34]. Intravenous injection of dissolved ozone might enhance humoral immunity [42,43]. However, one short-term time-series study observed that O_3_ was associated with an increased risk of TB [14], while another study observed opposite results [15]. Since O_3_ can react with other components in the air [11], the monitor might not be appropriate to measure individual O_3_ exposure [44]. Therefore, more studies are needed to explore and confirm the effect of O_3_.

The stratified analysis showed that male patients were more likely to be affected by air pollutant exposure, elderly patients were only more likely to be affected by NO_2_ and PM_2.5_ exposure, and patients were more likely to be affected by SO_2_ and PM_2.5_ in the cold season, and by CO in the warm season. With aging, the innate immune response decreases among the elderly [45]. Compared with women, men have a higher frequency of smoking or alcohol drinking, and both are risk factors for tuberculosis [46]. Furthermore, males are more likely to have respiratory infection-induced inflammation due to the immunosuppressive effect of testosterone [47]. Social contact patterns may also increase the burden of tuberculosis in men [48]. The modification of seasons varies among air pollutants. In the cold season, cold air may impair the nasal airway and bronchial mucosa and increase the risk of respiratory disease [49]. Air pollutants also diffuse slowly in the cold season, which may increase the inhalation of air pollutants, thereby increasing the effects of air pollutants exposure [50]. In the warm season, the cardiopulmonary load, respiratory rate, and inhalation volume may increase, which can also lead to increased inhalation of air pollutants [51].

There were some restrictions in this work. First, the air pollution level at the monitoring stations might not accurately reflect the air pollutant exposure level for individuals. Secondly, some important information on personal characteristics, such as smoking, drinking, and financial situation, was not available, and the associated confounding effects could not be adjusted for.

## 5. Conclusions

This study showed that short-term exposure to SO_2_, NO_2_, CO, and PM_2.5_, but not to O_3,_ were positively associated with TB incidence. Male and elderly patients were more likely to be affected by air pollutants. Seasons also had an impact on the effect of air pollutants exposure. Air pollutant exposure may be a risk factor for TB incidence.

## Figures and Tables

**Table 1:** Summary statistics for daily air pollutants and meteorological data in Xuhui District, Shanghai, 2014–2019.

Variables	Mean ± SD	Minimum	P25	P50	P75	Maximum	IQR
**Air pollutant**							
SO_2_ (μg/m^3^)	12.06 ± 8.11	2	7	10	15	90	8
NO_2_ (μg/m^3^)	45.76 ± 20.24	4	31	42	57	146	26
PM_2.5_ (μg/m^3^)	42.77 ± 28.58	3	23	36	55	232	32
CO (mg/m^3^)	0.81 ± 0.30	0.108	0.604	0.765	0.951	2.523	0.347
O_3_ (μg/m^3^)	70.57 ± 29.98	8	48	69	89	192	41
**Meteorological factors**							
Temperature (°C)	17.74 ± 8.57	−6.15	10.23	18.88	24.66	34.76	14.44
Air pressure (mmHg)	762.15 ± 6.81	741.44	756.41	762.23	767.49	779.88	11.08
Relative humidity (%)	73.27 ± 12.26	28.63	65.13	74.25	82.50	100.00	17.38
Wind velocity (m/s)	2.54 ± 0.89	0.50	1.88	2.38	3.00	6.88	1.13

SD: standard deviation; P: percentile; IQR: interquartile range.

**Table 2:** Results of correlation analysis between air pollutants and meteorological factors in Xuhui District, Shanghai, 2014–2019.

	PM_2.5_	SO_2_	NO_2_	CO	O_3_	Temperature	Air Pressure	Relative Humidity	Wind Velocity
PM_2.5_	1								
SO_2_	0.5830 *	1							
NO_2_	0.6630 *	0.5420 *	1						
CO	0.6376 *	0.4931 *	0.5087 *	1					
O_3_	−0.0608 *	−0.1401 *	−0.4191 *	−0.1783 *	1				
Temperature	−0.2953 *	−0.4335 *	−0.4841 *	−0.2471 *	0.5030 *	1			
Air pressure	0.1954 *	0.4311 *	0.4218 *	0.1818 *	−0.4327 *	−0.8861 *	1		
Relative humidity	−0.1593 *	−0.4377 *	−0.0809 *	−0.0536 *	−0.2613 *	0.1842 *	−0.3208 *	1	
Wind velocity	−0.3409 *	−0.1191 *	−0.5402 *	−0.2252 *	0.1005 *	0.0011	−0.0142	−0.0776 *	1

SO_2_: sulfur dioxide; NO_2_: nitrogen dioxide; PM_2.5_: particulate matter with an aerodynamic diameter less than 2.5 μm; CO: carbon monoxide; O_3_: ozone. *: *p* < 0.05.

## Data Availability

Restrictions apply to the availability of these data. Data was obtained from the Xuhui Center for Disease Control and Prevention and the China Environmental Monitoring Station at Xuhui Station, and are available from Y.Z. (ybzhou@fudan.edu.cn) with the permission of the Xuhui Center for Disease Control and Prevention and the China Environmental Monitoring Station at Xuhui Station.

## Supplementary Materials

The following supporting information can be downloaded at: https://www.mdpi.com/article/10.3390/ijerph19106085/s1, Tables S1–S5: The effect modification of gender, age, and season for SO_2_, NO_2_, PM_2.5_, CO, and O_3_; Tables S6–S10: The sensitivity analysis of the multipollutant model for SO_2_, NO_2_, PM_2.5_, CO, and O_3_; Tables S11–S15: The sensitivity analysis of the changes for meteorological factors (1–5) for SO_2_, NO_2_, PM_2.5_, CO, and O_3_; Tables S16–S20: The sensitivity analysis of the different lag days to test the delayed effects of SO_2_, NO_2_, PM_2.5_, CO, and O_3_.
