# Supplementary material for: Association of Daily Exposure to Air Pollutants with the Risk of Tuberculosis in Xuhui District of Shanghai, China
Source: Int J Environ Res Public Health. 2022 May 17;19(10):6085. doi: 10.3390/ijerph19106085 (PMC9141396; doi:10.3390/ijerph19106085)
Supplement: Supplementary file 1 [file ijerph-19-06085-s001.zip › ijerph-1698148-supplementary.pdf]

Supplementary Materials

Association of Daily Exposure to Air Pollutants with the Risk of Tuberculosis in Xuhui District of Shanghai, China

Ying Xiong [1,2,3], Meixia Yang [4,*], Zhengzhong Wang [1,2,3], Honglin Jiang [1,2,3], Ning Xu [1,2,3], Yixin Tong [1,2,3], Jiangfan Yin [1,2,3], Yue Chen [5], Qingwu Jiang [1,2,3] and Yibiao Zhou [1,2,3,*]

[1] School of Public Health, Fudan University, Building 8, 130 Dong'an Road, Shanghai 200032, China; 20211020140@fudan.edu.cn (Y.X.); 20211020023@fudan.edu.cn (Z.W.); hljiang20@fudan.edu.cn (H.J.); 21111020043@m.fudan.edu.cn (N.X.); 21211020016@m.fudan.edu.cn (Y.T.); 21211020154@m.fudan.edu.cn (J.Y.); jiangqw@fudan.edu.cn (Q.J.)

[2] Key Laboratory of Public Health Safety, Fudan University, Ministry of Education, Building 8, 130 Dong'an Road, Shanghai 200032, China

[3] Center for Tropical Disease Research, Fudan University, Building 8, 130 Dong'an Road, Shanghai 200032, China

[4] Xuhui Center for Disease Control and Prevention, Shanghai 200237, China

[5] School of Epidemiology and Public Health, Faculty of Medicine, University of Ottawa, 451 Smyth Road, Ottawa, ON K1H 8M5, Canada; ychen@uottawa.ca

* Correspondence: ymeixia@sina.com (M.Y.); ybzhou@fudan.edu.cn (Y.Z.)

1. Results of the analyses of the modification effect.

Table S1. The effect modification between sulfur dioxide (SO_2) and TB cases by gender, age, and season.

	Gender	Age	Season
Difference	-0.057(-0.151, 0.037)	-0.058(-0.195, 0.079)	-0.032(-0.108, 0.044)

Table S2. The effect modification between nitrogen dioxide (NO_2) and TB cases by gender, age, and season.

	Gender	Age	Season
Difference	-0.023(-0.118, 0.072)	-0.074(-0.184, 0.036)	0.004(-0.091, 0.099)

Table S3. The effect modification between particulate matter with an aerodynamic diameter less than 2.5 μm ($PM_{2.5}$) and TB cases by gender, age, and season.

	Gender	Age	Season
Difference	-0.124(-0.251, 0.003)	-0.183(-0.346, -0.020)	-0.311(-0.510, -0.112)

Table S4. The effect modification between carbon monoxide (CO) and TB cases by gender, age, and season.

	Gender	Age	Season
Difference	-0.042(-0.093, 0.009)	0.051(-0.009, 0.111)	0.105(0.011, 0.199)

Table S5. The effect modification between ozone (O_3) and TB cases by gender, age, and season.

	Gender	Age	Season
Difference	0.021(-0.045, 0.087)	-0.002(-0.084, 0.080)	-0.013(-0.107, 0.081)

2. Sensitivity analyses

Table S6. The sensitivity analysis about the multipollutant model for SO_2.

Lag	Lag-specific relative risk			Cumulative relative risk		
	RR	LCI	UCI	RR	LCI	UCI
lag0	1.011	0.931	1.098	1.011	0.931	1.098
lag1	1.048	0.997	1.101	1.059	0.932	1.204
lag2	**1.071**	**1.026**	**1.118**	1.134	0.972	1.324
lag3	**1.077**	**1.03**	**1.126**	**1.222**	**1.023**	**1.46**
lag4	**1.071**	**1.027**	**1.117**	**1.308**	**1.068**	**1.602**
lag5	**1.058**	**1.02**	**1.098**	**1.385**	**1.103**	**1.739**
lag6	**1.045**	**1.012**	**1.079**	**1.447**	**1.126**	**1.86**
lag7	**1.034**	**1.003**	**1.065**	**1.496**	**1.139**	**1.964**
lag8	1.024	0.995	1.054	**1.532**	**1.144**	**2.052**
lag9	1.016	0.988	1.046	**1.557**	**1.142**	**2.124**
lag10	1.01	0.982	1.039	**1.573**	**1.133**	**2.184**
lag11	1.005	0.978	1.033	**1.581**	**1.119**	**2.232**
lag12	1.001	0.975	1.028	**1.583**	**1.102**	**2.272**
lag13	0.998	0.974	1.023	**1.58**	**1.084**	**2.304**
lag14	0.997	0.974	1.02	**1.575**	**1.064**	**2.331**
lag15	0.996	0.975	1.017	**1.568**	**1.046**	**2.352**
lag16	0.996	0.976	1.015	**1.561**	**1.029**	**2.37**
lag17	0.996	0.977	1.015	**1.555**	**1.014**	**2.385**
lag18	0.997	0.977	1.017	**1.55**	**1.001**	**2.399**
lag19	0.998	0.976	1.02	1.547	0.992	2.414
lag20	0.999	0.974	1.025	1.546	0.984	2.43
lag21	1.001	0.972	1.031	1.548	0.978	2.45

RR: relative risk; UCI: upper confidence interval; LCI: lower confidence interval; SO_2: sulfur dioxide.

Bold: $P<0.05$

Table S7. The sensitivity analysis about the multipollutant model for NO_2.

Lag	Lag-specific relative risk			Cumulative relative risk		
	RR	LCI	UCI	RR	LCI	UCI
lag0	1.003	0.925	1.088	1.003	0.925	1.088
lag1	1.026	0.976	1.077	1.029	0.907	1.167
lag2	1.043	0.996	1.091	1.073	0.921	1.25
lag3	**1.053**	**1.003**	**1.105**	1.129	0.944	1.35
lag4	**1.057**	**1.01**	**1.107**	1.193	0.97	1.469
lag5	**1.057**	**1.015**	**1.101**	1.262	0.996	1.599
lag6	**1.056**	**1.017**	**1.096**	**1.332**	**1.023**	**1.734**
lag7	**1.053**	**1.015**	**1.092**	**1.402**	**1.05**	**1.874**
lag8	**1.049**	**1.011**	**1.089**	**1.471**	**1.074**	**2.016**
lag9	**1.044**	**1.006**	**1.085**	**1.537**	**1.094**	**2.159**
lag10	1.039	1	1.079	**1.596**	**1.108**	**2.301**
lag11	1.033	0.994	1.073	**1.648**	**1.114**	**2.438**
lag12	1.026	0.988	1.065	**1.69**	**1.113**	**2.566**
lag13	1.018	0.982	1.056	**1.721**	**1.104**	**2.68**
lag14	1.01	0.975	1.046	**1.738**	**1.088**	**2.776**
lag15	1.001	0.968	1.036	**1.74**	**1.063**	**2.847**
lag16	0.992	0.96	1.026	**1.727**	**1.032**	**2.89**
lag17	0.983	0.951	1.017	1.698	0.993	2.904
lag18	0.974	0.939	1.01	1.654	0.948	2.887
lag19	0.965	0.927	1.004	1.596	0.896	2.842
lag20	0.955	0.913	0.999	1.524	0.838	2.771
lag21	0.946	0.899	0.995	1.441	0.775	2.68

RR: relative risk; UCI: upper confidence interval; LCI: lower confidence interval; NO_2: nitrogen dioxide.
Bold: $P<0.05$

Table S8. The sensitivity analysis about the multipollutant model for $PM_{2.5}$.

Lag	Lag-specific relative risk			Cumulative relative risk		
	RR	LCI	UCI	RR	LCI	UCI
lag0	0.966	0.81	1.152	0.966	0.81	1.152
lag1	1.015	0.918	1.122	0.981	0.749	1.284
lag2	1.051	0.971	1.137	1.03	0.752	1.413
lag3	1.067	0.983	1.159	1.1	0.774	1.562
lag4	1.071	0.99	1.158	1.177	0.798	1.738
lag5	1.066	0.995	1.142	1.255	0.816	1.931
lag6	1.06	0.995	1.128	1.33	0.83	2.132
lag7	1.055	0.992	1.121	1.403	0.841	2.34
lag8	1.051	0.988	1.117	1.474	0.849	2.558
lag9	1.048	0.984	1.116	1.544	0.854	2.791
lag10	1.046	0.981	1.114	1.614	0.857	3.041
lag11	1.044	0.98	1.113	1.686	0.858	3.314
lag12	1.044	0.981	1.111	1.76	0.858	3.611
lag13	1.044	0.983	1.109	1.838	0.859	3.934
lag14	1.045	0.987	1.108	1.922	0.861	4.286
lag15	1.047	0.99	1.107	2.012	0.867	4.668
lag16	1.049	0.992	1.109	2.11	0.875	5.084
lag17	1.051	0.993	1.113	2.218	0.888	5.539
lag18	1.054	0.991	1.121	2.337	0.904	6.043
lag19	1.057	0.986	1.132	2.47	0.923	6.609
lag20	1.06	0.98	1.147	2.618	0.944	7.26
lag21	1.063	0.972	1.163	2.784	0.965	8.029

RR: relative risk; UCI: upper confidence interval; LCI: lower confidence interval; $PM_{2.5}$: particulate matter less than 2.5 μm in aerodynamic diameter.

Bold: $P<0.05$

Table S9. The sensitivity analysis about the multipollutant model for CO.

Lag	Lag-specific relative risk			Cumulative relative risk		
	RR	LCI	UCI	RR	LCI	UCI
lag0	1.004	0.93	1.084	1.004	0.93	1.084
lag1	1.011	0.968	1.055	1.015	0.904	1.139
lag2	1.017	0.98	1.055	1.032	0.902	1.182
lag3	1.022	0.982	1.063	1.055	0.907	1.227
lag4	1.025	0.988	1.064	1.082	0.912	1.282
lag5	1.028	0.996	1.06	1.112	0.92	1.343
lag6	**1.029**	**1.002**	**1.058**	1.145	0.931	1.407
lag7	**1.03**	**1.004**	**1.057**	1.179	0.944	1.473
lag8	**1.03**	**1.004**	**1.058**	1.215	0.959	1.54
lag9	**1.03**	**1.003**	**1.058**	1.251	0.973	1.61
lag10	**1.029**	**1.001**	**1.057**	1.287	0.986	1.682
lag11	1.027	0.999	1.056	1.322	0.996	1.754
lag12	1.025	0.997	1.053	**1.355**	**1.004**	**1.827**
lag13	1.022	0.995	1.049	**1.385**	**1.01**	**1.899**
lag14	1.019	0.993	1.045	**1.411**	**1.012**	**1.967**
lag15	1.016	0.991	1.041	**1.433**	**1.011**	**2.03**
lag16	1.012	0.987	1.037	**1.45**	**1.008**	**2.086**
lag17	1.008	0.982	1.034	1.461	1	2.134
lag18	1.004	0.975	1.033	1.466	0.989	2.175
lag19	0.999	0.967	1.033	1.466	0.973	2.208
lag20	0.995	0.958	1.033	1.458	0.952	2.235
lag21	0.991	0.948	1.035	1.445	0.924	2.259

RR: relative risk; UCI: upper confidence interval; LCI: lower confidence interval; CO: carbon monoxide.

Bold: P<0.05

Table S10. The sensitivity analysis about the multipollutant model for O_3.

Lag	Lag-specific relative risk			Cumulative relative risk		
	RR	LCI	UCI	RR	LCI	UCI
lag0	1.032	0.975	1.092	1.032	0.975	1.092
lag1	1.007	0.976	1.039	1.039	0.955	1.131
lag2	0.991	0.965	1.019	1.03	0.933	1.137
lag3	0.985	0.957	1.015	1.015	0.909	1.133
lag4	0.986	0.959	1.014	1.001	0.884	1.133
lag5	0.991	0.968	1.014	0.991	0.864	1.138
lag6	0.995	0.975	1.016	0.987	0.848	1.148
lag7	0.999	0.979	1.019	0.985	0.837	1.16
lag8	1.001	0.981	1.022	0.986	0.829	1.174
lag9	1.002	0.981	1.024	0.989	0.821	1.19
lag10	1.002	0.981	1.024	0.991	0.814	1.207
lag11	1.002	0.98	1.023	0.993	0.806	1.223
lag12	1	0.979	1.021	0.993	0.796	1.238
lag13	0.998	0.978	1.018	0.99	0.785	1.249
lag14	0.994	0.976	1.014	0.985	0.772	1.257
lag15	0.991	0.973	1.01	0.976	0.756	1.258
lag16	0.987	0.968	1.005	0.963	0.739	1.254
lag17	0.982	0.963	1.002	0.945	0.719	1.243
lag18	**0.977**	**0.956**	**0.999**	0.924	0.697	1.225
lag19	**0.972**	**0.947**	**0.998**	0.898	0.672	1.202
lag20	**0.967**	**0.938**	**0.996**	0.869	0.643	1.174
lag21	**0.962**	**0.929**	**0.996**	0.836	0.611	1.143

RR: relative risk; UCI: upper confidence interval; LCI: lower confidence interval; O_3: ozone.

Bold: P<0.05

Table S11. The sensitivity analysis about the changes of *df* for meteorological factors (1-5) for SO_2.

	df=1			df=2			df=3			df=4			df=5		
Lag	RR	LCI	UCI	RR	LCI	UCI	RR	LCI	UCI	RR	LCI	UCI	RR	LCI	UCI
lag0	1.003	0.927	1.086	1.004	0.928	1.087	1.001	0.925	1.083	1.006	0.929	1.089	1.005	0.929	1.088
lag1	1.046	0.997	1.097	1.048	0.999	1.1	1.044	0.995	1.095	1.048	0.999	1.1	1.048	0.999	1.1
lag2	**1.074**	**1.031**	**1.119**	**1.077**	**1.034**	**1.123**	**1.072**	**1.028**	**1.118**	**1.076**	**1.033**	**1.121**	**1.076**	**1.032**	**1.121**
lag3	**1.082**	**1.037**	**1.129**	**1.086**	**1.04**	**1.133**	**1.081**	**1.035**	**1.129**	**1.084**	**1.038**	**1.131**	**1.083**	**1.038**	**1.131**
lag4	**1.076**	**1.034**	**1.12**	**1.08**	**1.037**	**1.124**	**1.076**	**1.032**	**1.121**	**1.077**	**1.035**	**1.122**	**1.077**	**1.034**	**1.122**
lag5	**1.063**	**1.026**	**1.101**	**1.066**	**1.029**	**1.104**	**1.063**	**1.026**	**1.101**	**1.064**	**1.027**	**1.102**	**1.064**	**1.026**	**1.102**
lag6	**1.049**	**1.017**	**1.082**	**1.051**	**1.019**	**1.084**	**1.049**	**1.017**	**1.082**	**1.049**	**1.017**	**1.083**	**1.049**	**1.017**	**1.082**
lag7	**1.037**	**1.008**	**1.068**	**1.038**	**1.009**	**1.069**	**1.037**	**1.007**	**1.068**	**1.037**	**1.008**	**1.067**	**1.037**	**1.007**	**1.067**
lag8	1.027	0.999	1.056	1.028	1	1.057	1.027	0.998	1.057	1.027	0.999	1.056	1.026	0.998	1.055
lag9	1.019	0.991	1.047	1.019	0.992	1.047	1.019	0.99	1.049	1.018	0.991	1.047	1.018	0.99	1.046
lag10	1.012	0.985	1.04	1.012	0.985	1.04	1.013	0.984	1.042	1.011	0.984	1.039	1.011	0.984	1.039
lag11	1.007	0.981	1.034	1.006	0.98	1.033	1.007	0.979	1.036	1.006	0.98	1.033	1.006	0.979	1.032
lag12	1.003	0.978	1.028	1.002	0.977	1.028	1.003	0.976	1.031	1.002	0.977	1.027	1.001	0.976	1.027
lag13	0.999	0.976	1.023	0.999	0.976	1.023	1	0.975	1.027	0.999	0.975	1.023	0.998	0.975	1.023
lag14	0.997	0.976	1.02	0.997	0.975	1.019	0.999	0.975	1.023	0.997	0.975	1.019	0.997	0.975	1.019
lag15	0.996	0.976	1.017	0.996	0.976	1.017	0.998	0.975	1.02	0.996	0.975	1.016	0.996	0.975	1.016
lag16	0.996	0.977	1.015	0.996	0.977	1.015	0.997	0.976	1.018	0.995	0.976	1.015	0.995	0.976	1.015
lag17	0.996	0.977	1.015	0.996	0.978	1.015	0.998	0.978	1.018	0.996	0.977	1.015	0.996	0.977	1.015
lag18	0.996	0.977	1.016	0.997	0.978	1.017	0.998	0.978	1.019	0.997	0.977	1.017	0.997	0.977	1.017
lag19	0.997	0.975	1.02	0.998	0.977	1.021	1	0.977	1.022	0.998	0.976	1.02	0.998	0.976	1.02
lag20	0.998	0.973	1.024	1	0.975	1.026	1.001	0.976	1.027	0.999	0.974	1.025	0.999	0.974	1.026
lag21	1	0.97	1.03	1.002	0.972	1.032	1.002	0.973	1.033	1.001	0.971	1.031	1.001	0.971	1.032

RR: relative risk; UCI: upper confidence interval; LCI: lower confidence interval; SO_2: sulfur dioxide.

Bold: $P<0.05$

Table S12. The sensitivity analysis about the changes of *df* for meteorological factors (1-5) for NO_2.

Lag	df=1			df=2			df=3			df=4			df=5		
	RR	LCI	UCI	RR	LCI	UCI	RR	LCI	UCI	RR	LCI	UCI	RR	LCI	UCI
lag0	1.004	0.928	1.087	1.003	0.927	1.085	1.002	0.926	1.085	0.999	0.923	1.081	1	0.923	1.083
lag1	1.028	0.98	1.079	1.028	0.98	1.079	1.028	0.98	1.079	1.025	0.976	1.076	1.025	0.976	1.076
lag2	**1.046**	**1.001**	**1.094**	**1.047**	**1.002**	**1.095**	**1.047**	**1.001**	**1.095**	1.044	0.998	1.092	1.044	0.998	1.092
lag3	**1.056**	**1.007**	**1.107**	**1.058**	**1.009**	**1.109**	**1.057**	**1.009**	**1.109**	**1.055**	**1.006**	**1.106**	**1.054**	**1.005**	**1.106**
lag4	**1.059**	**1.012**	**1.107**	**1.061**	**1.015**	**1.11**	**1.061**	**1.015**	**1.11**	**1.059**	**1.012**	**1.108**	**1.058**	**1.012**	**1.107**
lag5	**1.058**	**1.017**	**1.1**	**1.061**	**1.02**	**1.104**	**1.061**	**1.019**	**1.104**	**1.059**	**1.017**	**1.102**	**1.058**	**1.017**	**1.102**
lag6	**1.055**	**1.017**	**1.093**	**1.058**	**1.021**	**1.097**	**1.058**	**1.02**	**1.097**	**1.056**	**1.018**	**1.096**	**1.056**	**1.018**	**1.096**
lag7	**1.051**	**1.014**	**1.088**	**1.055**	**1.018**	**1.093**	**1.055**	**1.018**	**1.093**	**1.053**	**1.016**	**1.092**	**1.053**	**1.016**	**1.092**
lag8	**1.046**	**1.009**	**1.084**	**1.05**	**1.013**	**1.089**	**1.05**	**1.013**	**1.089**	**1.049**	**1.011**	**1.088**	**1.049**	**1.011**	**1.088**
lag9	**1.041**	**1.004**	**1.08**	**1.045**	**1.008**	**1.085**	**1.045**	**1.007**	**1.085**	**1.044**	**1.006**	**1.084**	**1.044**	**1.006**	**1.084**
lag10	1.036	0.998	1.075	**1.04**	**1.002**	**1.079**	**1.04**	**1.002**	**1.08**	1.039	1	1.079	1.039	1	1.079
lag11	1.03	0.992	1.068	1.034	0.996	1.073	1.034	0.996	1.073	1.033	0.994	1.073	1.033	0.994	1.073
lag12	1.023	0.986	1.061	1.027	0.99	1.065	1.027	0.99	1.065	1.026	0.989	1.065	1.026	0.988	1.065
lag13	1.016	0.981	1.053	1.02	0.984	1.056	1.02	0.984	1.056	1.019	0.983	1.056	1.019	0.983	1.056
lag14	1.009	0.975	1.044	1.012	0.978	1.047	1.012	0.978	1.047	1.011	0.977	1.047	1.011	0.977	1.047
lag15	1.001	0.969	1.035	1.004	0.972	1.038	1.004	0.972	1.038	1.003	0.97	1.038	1.003	0.97	1.038
lag16	0.994	0.962	1.026	0.996	0.964	1.029	0.996	0.964	1.029	0.995	0.963	1.029	0.995	0.963	1.029
lag17	0.986	0.953	1.019	0.988	0.955	1.021	0.988	0.955	1.021	0.987	0.954	1.021	0.987	0.954	1.021
lag18	0.978	0.943	1.013	0.979	0.945	1.015	0.979	0.945	1.014	0.978	0.944	1.014	0.978	0.944	1.014
lag19	0.969	0.932	1.008	0.971	0.933	1.01	0.97	0.933	1.009	0.97	0.932	1.009	0.97	0.932	1.009
lag20	0.961	0.92	1.005	0.962	0.92	1.006	0.962	0.92	1.005	0.961	0.919	1.005	0.961	0.919	1.005
lag21	0.953	0.906	1.003	0.954	0.907	1.003	0.953	0.906	1.002	0.952	0.905	1.002	0.952	0.905	1.002

RR: relative risk; UCI: upper confidence interval; LCI: lower confidence interval; NO_2: nitrogen dioxide.

Bold: $P<0.05$

Table S13. The sensitivity analysis about the changes of *df* for meteorological factors (1-5) for $PM_{2.5}$.

	df=1			df=2			df=3			df=4			df=5		
Lag	RR	LCI	UCI	RR	LCI	UCI	RR	LCI	UCI	RR	LCI	UCI	RR	LCI	UCI
lag0	0.997	0.861	1.156	0.99	0.853	1.148	0.991	0.854	1.15	0.995	0.857	1.155	1	0.861	1.161
lag1	1.034	0.948	1.126	1.027	0.941	1.12	1.027	0.941	1.121	1.03	0.943	1.125	1.034	0.946	1.129
lag2	1.06	0.982	1.143	1.054	0.976	1.138	1.054	0.975	1.138	1.055	0.977	1.14	1.058	0.979	1.143
lag3	1.072	0.989	1.162	1.067	0.984	1.157	1.067	0.983	1.157	1.067	0.984	1.158	1.069	0.985	1.161
lag4	1.075	0.996	1.16	1.071	0.991	1.156	1.07	0.99	1.156	1.07	0.99	1.156	1.071	0.991	1.158
lag5	**1.072**	**1.002**	**1.146**	1.068	0.998	1.143	1.068	0.997	1.143	1.066	0.996	1.142	1.068	0.997	1.144
lag6	**1.067**	**1.004**	**1.134**	**1.064**	**1.001**	**1.131**	1.064	1	1.132	1.062	0.998	1.13	1.064	0.999	1.132
lag7	**1.063**	**1.002**	**1.128**	1.061	1	1.126	1.061	0.999	1.127	1.059	0.996	1.125	1.06	0.998	1.127
lag8	1.06	0.999	1.126	1.059	0.997	1.124	1.058	0.996	1.125	1.056	0.993	1.123	1.057	0.994	1.125
lag9	1.058	0.995	1.124	1.057	0.994	1.123	1.056	0.992	1.124	1.054	0.99	1.122	1.055	0.991	1.124
lag10	1.056	0.992	1.123	1.055	0.992	1.122	1.055	0.99	1.123	1.052	0.988	1.121	1.054	0.989	1.123
lag11	1.054	0.991	1.121	1.054	0.991	1.121	1.053	0.989	1.122	1.051	0.987	1.12	1.053	0.988	1.122
lag12	1.053	0.991	1.119	1.053	0.991	1.119	1.053	0.99	1.119	1.051	0.988	1.118	1.053	0.989	1.12
lag13	1.052	0.993	1.116	1.053	0.993	1.116	1.052	0.992	1.117	1.051	0.99	1.116	1.053	0.992	1.118
lag14	1.052	0.995	1.113	1.053	0.996	1.114	1.052	0.994	1.114	1.052	0.993	1.114	1.053	0.995	1.116
lag15	1.052	0.997	1.111	1.053	0.998	1.112	1.053	0.996	1.112	1.053	0.996	1.112	1.054	0.997	1.114
lag16	1.053	0.997	1.111	1.054	0.999	1.112	1.053	0.997	1.112	1.054	0.998	1.113	1.056	0.999	1.115
lag17	1.053	0.996	1.114	1.055	0.998	1.115	1.054	0.996	1.115	1.055	0.997	1.117	1.057	0.999	1.119
lag18	1.054	0.992	1.12	1.056	0.994	1.122	1.055	0.992	1.121	1.057	0.995	1.124	1.059	0.996	1.126
lag19	1.055	0.986	1.129	1.057	0.988	1.131	1.056	0.986	1.13	1.059	0.989	1.134	1.061	0.99	1.136
lag20	1.056	0.977	1.141	1.058	0.98	1.143	1.057	0.978	1.142	1.061	0.982	1.147	1.063	0.983	1.149
lag21	1.057	0.968	1.155	1.059	0.97	1.157	1.058	0.968	1.155	1.063	0.973	1.162	1.065	0.974	1.164

RR: relative risk; UCI: upper confidence interval; LCI: lower confidence interval; $PM_{2.5}$: particulate matter less than 2.5 μm in aerodynamic diameter.

Bold: P<0.05

Table S14. The sensitivity analysis about the changes of *df* for meteorological factors (1-5) for CO.

	df=1			df=2			df=3			df=4			df=5		
Lag	RR	LCI	UCI	RR	LCI	UCI	RR	LCI	UCI	RR	LCI	UCI	RR	LCI	UCI
lag0	1.007	0.935	1.086	1.009	0.936	1.087	1.012	0.939	1.091	1.011	0.938	1.09	1.011	0.938	1.09
lag1	1.01	0.968	1.053	1.01	0.969	1.054	1.013	0.971	1.057	1.014	0.972	1.057	1.014	0.972	1.058
lag2	1.013	0.977	1.05	1.013	0.977	1.051	1.016	0.979	1.054	1.017	0.98	1.055	1.017	0.981	1.055
lag3	1.017	0.978	1.057	1.017	0.978	1.057	1.019	0.98	1.06	1.021	0.981	1.061	1.021	0.982	1.062
lag4	1.021	0.985	1.059	1.021	0.984	1.059	1.022	0.986	1.06	1.025	0.988	1.063	1.025	0.988	1.064
lag5	1.025	0.994	1.057	1.025	0.994	1.057	1.026	0.995	1.058	1.028	0.997	1.06	1.029	0.998	1.061
lag6	**1.028**	**1.002**	**1.056**	**1.028**	**1.001**	**1.055**	**1.028**	**1.002**	**1.056**	**1.031**	**1.004**	**1.059**	**1.032**	**1.005**	**1.06**
lag7	**1.031**	**1.005**	**1.057**	**1.029**	**1.004**	**1.056**	**1.03**	**1.004**	**1.056**	**1.033**	**1.007**	**1.059**	**1.034**	**1.008**	**1.06**
lag8	**1.032**	**1.006**	**1.058**	**1.03**	**1.004**	**1.057**	**1.03**	**1.005**	**1.057**	**1.033**	**1.007**	**1.06**	**1.034**	**1.008**	**1.061**
lag9	**1.032**	**1.005**	**1.059**	**1.03**	**1.003**	**1.058**	**1.03**	**1.003**	**1.058**	**1.033**	**1.006**	**1.061**	**1.034**	**1.007**	**1.062**
lag10	**1.031**	**1.003**	**1.059**	**1.029**	**1.002**	**1.057**	**1.029**	**1.001**	**1.057**	**1.032**	**1.004**	**1.06**	**1.033**	**1.005**	**1.062**
lag11	**1.029**	**1.002**	**1.057**	1.027	1	1.056	1.027	0.999	1.055	**1.03**	**1.002**	**1.058**	**1.031**	**1.003**	**1.06**
lag12	1.027	1	1.054	1.025	0.998	1.053	1.024	0.997	1.052	1.027	1	1.055	1.028	1	1.056
lag13	1.023	0.997	1.05	1.022	0.995	1.048	1.021	0.995	1.048	1.024	0.997	1.051	1.025	0.998	1.052
lag14	1.02	0.995	1.045	1.018	0.993	1.043	1.017	0.992	1.043	1.02	0.994	1.046	1.021	0.995	1.047
lag15	1.015	0.991	1.04	1.013	0.989	1.038	1.013	0.989	1.038	1.015	0.99	1.04	1.016	0.991	1.041
lag16	1.011	0.987	1.035	1.009	0.984	1.033	1.008	0.984	1.033	1.01	0.986	1.035	1.011	0.987	1.036
lag17	1.006	0.98	1.031	1.004	0.978	1.03	1.003	0.978	1.029	1.005	0.979	1.031	1.006	0.98	1.032
lag18	1	0.973	1.029	0.998	0.971	1.027	0.998	0.97	1.027	0.999	0.971	1.028	1	0.972	1.029
lag19	0.995	0.963	1.027	0.993	0.961	1.025	0.993	0.961	1.025	0.994	0.962	1.027	0.995	0.963	1.028
lag20	0.989	0.953	1.026	0.987	0.951	1.024	0.987	0.951	1.024	0.988	0.952	1.026	0.989	0.953	1.027
lag21	0.983	0.942	1.026	0.981	0.941	1.024	0.982	0.94	1.024	0.982	0.941	1.025	0.983	0.942	1.026

RR: relative risk; UCI: upper confidence interval; LCI: lower confidence interval; CO: carbon monoxide.

Bold: $P<0.05$

Table S15. The sensitivity analysis about the changes of *df* for meteorological factors (1-5) for O_3.

	df=1			df=2			df=3			df=4			df=5		
Lag	RR	LCI	UCI	RR	LCI	UCI	RR	LCI	UCI	RR	LCI	UCI	RR	LCI	UCI
lag0	1.02	0.965	1.077	1.021	0.966	1.079	1.025	0.97	1.083	1.025	0.969	1.083	1.025	0.97	1.084
lag1	1.001	0.971	1.032	1.002	0.972	1.034	1.004	0.974	1.036	1.004	0.974	1.036	1.005	0.974	1.036
lag2	0.99	0.963	1.016	0.991	0.964	1.017	0.991	0.965	1.018	0.991	0.965	1.018	0.991	0.965	1.018
lag3	0.986	0.958	1.015	0.987	0.959	1.016	0.987	0.959	1.016	0.987	0.959	1.016	0.987	0.959	1.016
lag4	0.988	0.961	1.015	0.988	0.962	1.016	0.989	0.962	1.016	0.988	0.962	1.015	0.988	0.962	1.016
lag5	0.992	0.969	1.015	0.993	0.97	1.016	0.993	0.97	1.016	0.993	0.97	1.016	0.993	0.97	1.016
lag6	0.997	0.977	1.017	0.997	0.977	1.017	0.997	0.977	1.018	0.997	0.977	1.017	0.997	0.977	1.018
lag7	1	0.981	1.019	1	0.981	1.02	1.001	0.981	1.021	1	0.981	1.02	1	0.981	1.02
lag8	1.002	0.982	1.022	1.002	0.983	1.023	1.003	0.983	1.023	1.002	0.983	1.023	1.003	0.983	1.023
lag9	1.003	0.983	1.024	1.004	0.983	1.024	1.004	0.983	1.025	1.003	0.983	1.024	1.004	0.983	1.025
lag10	1.003	0.982	1.025	1.004	0.983	1.025	1.004	0.983	1.026	1.003	0.982	1.025	1.004	0.983	1.025
lag11	1.003	0.982	1.024	1.003	0.982	1.024	1.003	0.982	1.025	1.003	0.982	1.024	1.003	0.982	1.024
lag12	1.001	0.981	1.022	1.001	0.981	1.022	1.002	0.981	1.022	1.001	0.98	1.022	1.001	0.981	1.022
lag13	0.999	0.979	1.019	0.999	0.979	1.019	0.999	0.98	1.019	0.998	0.979	1.018	0.999	0.979	1.019
lag14	0.996	0.977	1.015	0.996	0.977	1.015	0.996	0.977	1.015	0.995	0.977	1.014	0.996	0.977	1.015
lag15	0.992	0.974	1.011	0.993	0.975	1.011	0.992	0.974	1.011	0.992	0.973	1.01	0.992	0.974	1.01
lag16	0.988	0.97	1.007	0.989	0.971	1.007	0.988	0.97	1.007	0.987	0.969	1.006	0.988	0.969	1.006
lag17	0.984	0.965	1.004	0.984	0.965	1.004	0.984	0.965	1.003	0.983	0.964	1.003	0.983	0.964	1.003
lag18	0.979	0.958	1.001	0.98	0.958	1.002	0.979	0.958	1.001	0.978	0.957	1	0.978	0.957	1
lag19	0.974	0.95	1	0.975	0.95	1	**0.974**	**0.949**	**0.999**	**0.973**	**0.948**	**0.998**	**0.973**	**0.948**	**0.998**
lag20	**0.969**	**0.941**	**0.999**	**0.97**	**0.942**	**0.999**	**0.969**	**0.94**	**0.998**	**0.968**	**0.939**	**0.997**	**0.968**	**0.939**	**0.997**
lag21	**0.964**	**0.932**	**0.998**	**0.965**	**0.932**	**0.999**	**0.963**	**0.931**	**0.997**	**0.963**	**0.93**	**0.996**	**0.962**	**0.93**	**0.996**

RR: relative risk; UCI: upper confidence interval; LCI: lower confidence interval; O_3: ozone.

Bold: P<0.05

Table S16. The sensitivity analysis about the different lag days to test the delayed effects of SO_2.

	lag=19 days			lag=21 days			lag=23 days		
Lag	RR	LCI	UCI	RR	LCI	UCI	RR	LCI	UCI
lag0	0.998	0.921	1.082	1.001	0.925	1.083	1.009	0.934	1.09
lag1	1.048	0.999	1.098	1.044	0.995	1.095	1.05	1	1.102
lag2	**1.078**	**1.035**	**1.124**	**1.072**	**1.028**	**1.118**	**1.078**	**1.035**	**1.123**
lag3	**1.085**	**1.04**	**1.133**	**1.081**	**1.035**	**1.129**	**1.088**	**1.043**	**1.135**
lag4	**1.076**	**1.035**	**1.119**	**1.076**	**1.032**	**1.121**	**1.085**	**1.04**	**1.131**
lag5	**1.06**	**1.026**	**1.095**	**1.063**	**1.026**	**1.101**	**1.073**	**1.033**	**1.115**
lag6	**1.045**	**1.016**	**1.074**	**1.049**	**1.017**	**1.082**	**1.06**	**1.024**	**1.096**
lag7	**1.032**	**1.006**	**1.058**	**1.037**	**1.007**	**1.068**	**1.047**	**1.015**	**1.081**
lag8	1.022	0.997	1.047	1.027	0.998	1.057	**1.037**	**1.006**	**1.069**
lag9	1.013	0.989	1.038	1.019	0.99	1.049	1.028	0.998	1.059
lag10	1.007	0.984	1.031	1.013	0.984	1.042	1.02	0.991	1.051
lag11	1.002	0.98	1.025	1.007	0.979	1.036	1.014	0.985	1.044
lag12	0.999	0.977	1.02	1.003	0.976	1.031	1.009	0.981	1.038
lag13	0.996	0.976	1.017	1	0.975	1.027	1.005	0.978	1.033
lag14	0.995	0.976	1.015	0.999	0.975	1.023	1.002	0.977	1.028
lag15	0.995	0.976	1.014	0.998	0.975	1.02	1	0.976	1.024
lag16	0.996	0.976	1.016	0.997	0.976	1.018	0.999	0.976	1.021
lag17	0.997	0.975	1.019	0.998	0.978	1.018	0.998	0.977	1.019
lag18	0.998	0.973	1.024	0.998	0.978	1.019	0.998	0.978	1.018
lag19	1	0.97	1.03	1	0.977	1.022	0.998	0.978	1.019
lag20				1.001	0.976	1.027	0.999	0.978	1.021
lag21				1.002	0.973	1.033	1	0.976	1.025
lag22							1.002	0.974	1.03
lag23							1.003	0.972	1.035

RR: relative risk; UCI: upper confidence interval; LCI: lower confidence interval; SO_2: sulfur dioxide.

Bold: $P<0.05$

Table S17. The sensitivity analysis about the different lag days to test the delayed effects of NO_2.

	lag=19 days			lag=21 days			lag=23 days		
Lag	RR	LCI	UCI	RR	LCI	UCI	RR	LCI	UCI
lag0	1.014	0.936	1.099	1.002	0.926	1.085	1.001	0.926	1.083
lag1	1.035	0.987	1.085	1.028	0.98	1.079	1.03	0.982	1.081
lag2	**1.05**	**1.003**	**1.1**	**1.047**	**1.001**	**1.095**	**1.051**	**1.007**	**1.098**
lag3	**1.06**	**1.01**	**1.112**	**1.057**	**1.009**	**1.109**	**1.062**	**1.014**	**1.112**
lag4	**1.064**	**1.018**	**1.112**	**1.061**	**1.015**	**1.11**	**1.064**	**1.016**	**1.113**
lag5	**1.065**	**1.025**	**1.107**	**1.061**	**1.019**	**1.104**	**1.06**	**1.018**	**1.105**
lag6	**1.065**	**1.027**	**1.103**	**1.058**	**1.02**	**1.097**	**1.054**	**1.016**	**1.094**
lag7	**1.063**	**1.025**	**1.102**	**1.055**	**1.018**	**1.093**	**1.048**	**1.012**	**1.086**
lag8	**1.059**	**1.021**	**1.099**	**1.05**	**1.013**	**1.089**	**1.042**	**1.006**	**1.08**
lag9	**1.055**	**1.016**	**1.096**	**1.045**	**1.007**	**1.085**	1.036	0.999	1.074
lag10	**1.05**	**1.011**	**1.09**	**1.04**	**1.002**	**1.08**	1.03	0.993	1.069
lag11	**1.044**	**1.006**	**1.083**	1.034	0.996	1.073	1.024	0.986	1.062
lag12	**1.037**	**1.001**	**1.075**	1.027	0.99	1.065	1.017	0.98	1.056
lag13	1.03	0.995	1.065	1.02	0.984	1.056	1.011	0.975	1.049
lag14	1.022	0.989	1.056	1.012	0.978	1.047	1.005	0.97	1.041
lag15	1.013	0.98	1.048	1.004	0.972	1.038	0.999	0.965	1.033
lag16	1.005	0.97	1.041	0.996	0.964	1.029	0.993	0.96	1.026
lag17	0.996	0.957	1.036	0.988	0.955	1.021	0.986	0.955	1.019
lag18	0.987	0.943	1.032	0.979	0.945	1.014	0.98	0.949	1.012
lag19	0.978	0.928	1.03	0.97	0.933	1.009	0.974	0.942	1.007
lag20				0.962	0.92	1.005	0.968	0.934	1.003
lag21				0.953	0.906	1.002	0.962	0.925	1
lag22							0.956	0.915	0.999
lag23							0.95	0.904	0.998

RR: relative risk; UCI: upper confidence interval; LCI: lower confidence interval; NO_2: nitrogen dioxide.

Bold: $P<0.05$

Table S18. The sensitivity analysis about the different lag days to test the delayed effects of $PM_{2.5}$.

	lag=19 days			lag=21 days			lag=23 days		
Lag	RR	LCI	UCI	RR	LCI	UCI	RR	LCI	UCI
lag0	0.988	0.849	1.15	0.991	0.854	1.15	0.997	0.861	1.154
lag1	1.024	0.939	1.117	1.027	0.941	1.121	1.032	0.945	1.128
lag2	1.048	0.966	1.137	1.054	0.975	1.138	1.059	0.983	1.142
lag3	1.058	0.972	1.151	1.067	0.983	1.157	1.074	0.992	1.162
lag4	1.057	0.979	1.142	1.07	0.99	1.156	1.079	0.998	1.166
lag5	1.052	0.986	1.124	1.068	0.997	1.143	**1.078**	**1.004**	**1.156**
lag6	1.047	0.986	1.112	1.064	1	1.132	**1.074**	**1.008**	**1.144**
lag7	1.043	0.982	1.107	1.061	0.999	1.127	**1.071**	**1.008**	**1.137**
lag8	1.039	0.977	1.105	1.058	0.996	1.125	**1.068**	**1.005**	**1.134**
lag9	1.037	0.974	1.103	1.056	0.992	1.124	**1.065**	**1.002**	**1.132**
lag10	1.034	0.972	1.101	1.055	0.99	1.123	1.063	0.999	1.131
lag11	1.033	0.972	1.098	1.053	0.989	1.122	1.061	0.997	1.13
lag12	1.032	0.974	1.094	1.053	0.99	1.119	1.06	0.996	1.128
lag13	1.032	0.976	1.091	1.052	0.992	1.117	1.059	0.996	1.125
lag14	1.032	0.977	1.09	1.052	0.994	1.114	1.058	0.997	1.122
lag15	1.032	0.976	1.091	1.053	0.996	1.112	1.057	0.998	1.119
lag16	1.033	0.972	1.097	1.053	0.997	1.112	1.057	1	1.117
lag17	1.033	0.965	1.106	1.054	0.996	1.115	1.056	1.001	1.116
lag18	1.034	0.955	1.12	1.055	0.992	1.121	1.056	1	1.116
lag19	1.035	0.944	1.135	1.056	0.986	1.13	1.056	0.998	1.119
lag20				1.057	0.978	1.142	1.057	0.993	1.124
lag21				1.058	0.968	1.155	1.057	0.987	1.131
lag22							1.057	0.979	1.141
lag23							1.057	0.971	1.152

RR: relative risk; UCI: upper confidence interval; LCI: lower confidence interval; $PM_{2.5}$: particulate matter less than 2.5 μm in aerodynamic diameter.

Bold: P<0.05

Table S19. The sensitivity analysis about the different lag days to test the delayed effects of CO.

Lag	lag=19 days			lag=21 days			lag=23 days		
	RR	LCI	UCI	RR	LCI	UCI	RR	LCI	UCI
lag0	1.011	0.936	1.091	1.012	0.939	1.091	1.014	0.942	1.092
lag1	1.008	0.967	1.051	1.013	0.971	1.057	1.018	0.975	1.063
lag2	1.008	0.97	1.047	1.016	0.979	1.054	1.021	0.986	1.058
lag3	1.012	0.972	1.054	1.019	0.98	1.06	1.023	0.986	1.063
lag4	1.018	0.982	1.055	1.022	0.986	1.06	1.025	0.988	1.063
lag5	1.023	0.994	1.054	1.026	0.995	1.058	1.026	0.994	1.059
lag6	**1.028**	**1.001**	**1.055**	**1.028**	**1.002**	**1.056**	1.027	0.999	1.055
lag7	**1.03**	**1.004**	**1.057**	**1.03**	**1.004**	**1.056**	**1.027**	**1.001**	**1.053**
lag8	**1.032**	**1.004**	**1.06**	**1.03**	**1.005**	**1.057**	**1.026**	**1.001**	**1.052**
lag9	**1.031**	**1.003**	**1.06**	**1.03**	**1.003**	**1.058**	1.026	1	1.052
lag10	**1.03**	**1.002**	**1.059**	**1.029**	**1.001**	**1.057**	1.025	0.998	1.052
lag11	1.027	1	1.056	1.027	0.999	1.055	1.024	0.997	1.051
lag12	1.024	0.997	1.051	1.024	0.997	1.052	1.022	0.995	1.05
lag13	1.019	0.994	1.045	1.021	0.995	1.048	1.02	0.994	1.048
lag14	1.014	0.989	1.039	1.017	0.992	1.043	1.018	0.993	1.045
lag15	1.008	0.983	1.034	1.013	0.989	1.038	1.016	0.991	1.042
lag16	1.002	0.974	1.03	1.008	0.984	1.033	1.014	0.99	1.039
lag17	0.995	0.964	1.027	1.003	0.978	1.029	1.012	0.988	1.036
lag18	0.988	0.952	1.026	0.998	0.97	1.027	1.009	0.985	1.034
lag19	0.981	0.939	1.026	0.993	0.961	1.025	1.006	0.98	1.033
lag20				0.987	0.951	1.024	1.003	0.975	1.032
lag21				0.982	0.94	1.024	1.001	0.969	1.033
lag22							0.998	0.962	1.035
lag23							0.995	0.954	1.037

RR: relative risk; UCI: upper confidence interval; LCI: lower confidence interval; CO: carbon monoxide.

pm

Bold: P<0.05

Table S20. The sensitivity analysis about the different lag days to test the delayed effects of O_3.

Lag	lag=19 days			lag=21 days			lag=23 days		
	RR	LCI	UCI	RR	LCI	UCI	RR	LCI	UCI
lag0	1.022	0.965	1.081	1.025	0.97	1.083	1.019	0.965	1.076
lag1	1.003	0.973	1.034	1.004	0.974	1.036	1.005	0.974	1.037
lag2	0.992	0.964	1.02	0.991	0.965	1.018	0.996	0.971	1.022
lag3	0.988	0.959	1.019	0.987	0.959	1.016	0.992	0.965	1.02
lag4	0.991	0.964	1.018	0.989	0.962	1.016	0.992	0.965	1.019
lag5	0.995	0.973	1.018	0.993	0.97	1.016	0.993	0.97	1.017
lag6	0.999	0.979	1.02	0.997	0.977	1.018	0.996	0.976	1.017
lag7	1.002	0.982	1.023	1.001	0.981	1.021	0.998	0.979	1.017
lag8	1.004	0.983	1.025	1.003	0.983	1.023	0.999	0.98	1.019
lag9	1.005	0.983	1.027	1.004	0.983	1.025	1	0.98	1.02
lag10	1.005	0.983	1.027	1.004	0.983	1.026	1	0.98	1.021
lag11	1.005	0.983	1.026	1.003	0.982	1.025	1	0.979	1.02
lag12	1.003	0.983	1.024	1.002	0.981	1.022	0.999	0.979	1.02
lag13	1.002	0.982	1.021	0.999	0.98	1.019	0.997	0.978	1.018
lag14	1	0.981	1.018	0.996	0.977	1.015	0.996	0.976	1.015
lag15	0.997	0.978	1.016	0.992	0.974	1.011	0.994	0.975	1.013
lag16	0.994	0.973	1.015	0.988	0.97	1.007	0.991	0.973	1.009
lag17	0.991	0.967	1.015	0.984	0.965	1.003	0.988	0.971	1.007
lag18	0.987	0.96	1.016	0.979	0.958	1.001	0.986	0.967	1.004
lag19	0.984	0.951	1.018	**0.974**	**0.949**	**0.999**	0.982	0.963	1.002
lag20				**0.969**	**0.94**	**0.998**	0.979	0.958	1.001
lag21				**0.963**	**0.931**	**0.997**	0.976	0.951	1.001
lag22							0.972	0.945	1.001
lag23							0.969	0.938	1.001

RR: relative risk; UCI: upper confidence interval; LCI: lower confidence interval; O_3: ozone.

Bold: P<0.05
